# Porous Hydroxyapatite and Aluminium-Oxide Ceramic Orbital Implant Evaluation Using CBCT Scanning: A Method for *In Vivo* Porous Structure Evaluation and Monitoring

**DOI:** 10.1155/2012/764749

**Published:** 2012-02-28

**Authors:** Olga Lukáts, Péter Bujtár, George K. Sándor, József Barabás

**Affiliations:** ^1^Department of Ophthalmology, Semmelweis University, Budapest 1083, Hungary; ^2^Department of Oral and Maxillofacial Surgery and Dentistry, Faculty of Dentistry, Semmelweis University, Budapest, Hungary; ^3^Oral and Maxillofacial Surgery, University of Leicester, Leicester, UK; ^4^Department of Tissue Engineering, Regea Institute for Regenerative Medicine, University of Tampere, 33520 Tampere, Finland; ^5^Department of Oral and Maxillofacial Surgery, University of Oulu, 90014 Oulu, Finland

## Abstract

*Objective*. This study aimed to define CBCT as a technique for postimplantation *in vivo* examination of porous hydroxyapatite and aluminium-oxide orbital implant shape, volume and density changes. *Methods and Materials*. CBCT was used to evaluate 30 enucleated patients treated with spherical polyglactin 910 wrapped hydroxyapatite and aluminum-oxide orbital implants. The mean duration of patient followup was 3.2 years or 1338 days with a range of 0.2 to 7.2 years or 79 to 2636 days in a population with an average age of 40.8 years. *Results*. The resolution of currently clinically used CBCT equipment allowed detailed structural observation of the orbital hydroxyapatite implants with some modifications. Volume and shape estimations were possible while density evaluation was more complicated compared to medical source computed tomography. The mean densities of the orbital implants were followed and a consistent gradual decrease identified from the beginning of implantation which was better defined after the applied correction procedure. *Conclusion*. CBCT with lower dosages of radiation exposure can be used to follow changes in implanted high-density porous structures. The density evaluation is possible with calibration modifications. Changes in orbital implant densities identified in this study may correspond to healing and maturation of soft tissues surrounding and penetrating the implants.

## 1. Introduction

Enucleation has long been used for the treatment of ocular diseases such as intraocular malignancy, severe trauma, and blind painful eye. The major purpose of enucleation is to remove the diseased globe intact and to provide a cosmetically acceptable appearance [1]. The first orbital removal for medical treatment was performed in 1583. In 1885 the first orbital implants to replace the obvious cosmetically deleterious volume loss after evisceration were hollow glass, gold, or silver spheres [1–3]. Since then numerous studies describing different types of enucleation techniques and various types of orbital implants have been published [4–10]. These implanted spheres are permanently buried within the soft tissues of the orbit. Later a cosmetically pleasing, removable shield-like ocular prosthesis made from glass or medical grade acrylic is placed between the remaining conjunctiva and eyelids and supported by the sphere-shaped orbital implant. 

The characteristics of an ideal orbital implant include adequate volume replacement of the lost globe, good motility and support transmitted to the overlying ocular prosthesis, low rate of complications, and with an economical cost. The orbital implant should be technically simple to implant, biocompatible, and nondegradable as the orbital implant is regarded as being permanent.

Porous orbital implants are preferred with porosity rates of 80% [11] and pore diameters of 150 *μ*m to 400 *μ*m which favour tissue ingrowth [12]. The most commonly used porous materials for spherical orbital implants include xenografts such as coralline, bovine bone scaffolds, or synthetic materials such as hydroxyapatite (HA), aluminium oxide (AO), and porous polyethylene [13–30]. HA has been used as a bone substitute in orthopaedics and oral and maxillofacial surgery since 1975. HA received FDA approval for use as an orbital implant in 1989 (Bio-Eye, Integrated Orbital Implants, San Diego, CA) [31].

There is little information about composition, density, volume, and shape changes of AO and HA implants in the enucleated orbit. When follow-up imaging is required then medical source computed tomography (MSCT) has been used [12–14]. MSCT has the disadvantage of a significant radiation dose with each examination.

Other imaging options include bone scans and magnetic resonance imaging (MRI). Bone scans require the intravenous injection of radionucleotide material while MRI scans are enhanced by the intravenous injection of gadolinium dye. Both modalities have great future potential.

Intraorbital HA implant examination with cone beam computed tomography (CBCT) with lower radiation exposure than MSCT may be a simpler and reliable clinical alternative to orbital implant monitoring and followup which has yet to be evaluated.

CBCT was described in 1982 [19] and recently applied to dentomaxillofacial diagnostics. This radiologic modality is an alternative instead of MSCT which is reliable for the detection of soft and hard tissues which are numerically described by Hounsfield units (HU).

In the past CBCT was less accurate than MSCT in HU density measurements so that only the quantitative estimation of the tissues was possible [20–26]. However recent CBCTs use higher isometric voxel resolution from 72 to 400 *μ*m. MSCT voxels are usually not isometric and larger than 250 *μ*m. Generally image noise is higher with CBCT [27], while the dose of radiation is much lower [21]. The field of view (FOV) of dental and maxillofacial CBCTs includes only a part of the head and neck region resulting in more artifact [28] while the FOV with MSCT can incorporate as much of the body as necessary. However, monitoring specific anatomic areas can be performed with CBCT using less radiation.

CBCT can be used for the preoperative evaluation of calcified bone-like structures. Attempts to compensate for the HU density distortion or inaccuracy have been resolved with conversion coefficients [29, 30]. CBCTs are less expensive than MSCT making CBCT suitable for even single dental offices or medical clinics [19, 21]. Moreover CBCT combined with other radiological modalities is another option.

CBCT may be potentially useful in tissue engineering paradigms which may require long-term monitoring of scaffolds in a noninvasive manner with the lowest possible doses of radiation. The enucleated orbit provides a unique model to evaluate porous scaffold material after implantation in the orbit, a cavity with varying tissue densities.

This study evaluates the possible role of CBCT in the post operative monitoring of the orbit, a specific and well-defined anatomic structure, with a porous AO and HA implant. Specifically the aim of this study was to examine orbital implants for shape, volumetric, and density changes following implantation.

## 2. Materials and Method

### 2.1. Surgical Procedure

**Figure 1:**
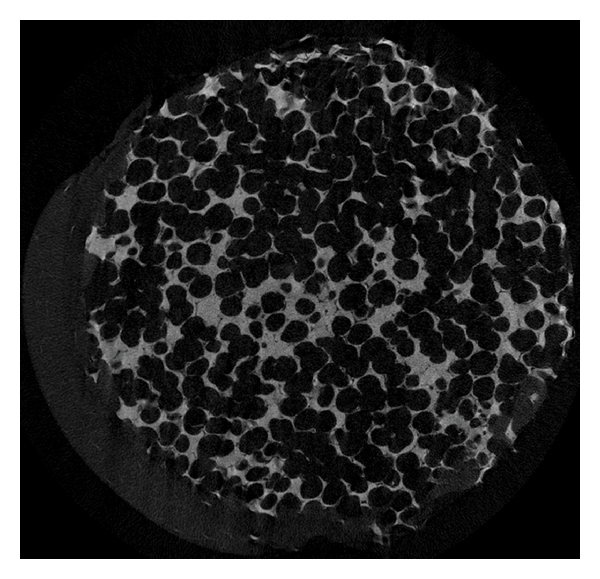
Microstructure of the HA orbital implant. A planar reconstruction of a native 18 mm-diameter HA orbital implant. Acquisition performed with microCT applying 7.815 micrometer voxel size (SkyScan, Kontich, Belgium).

**Figure 2:**
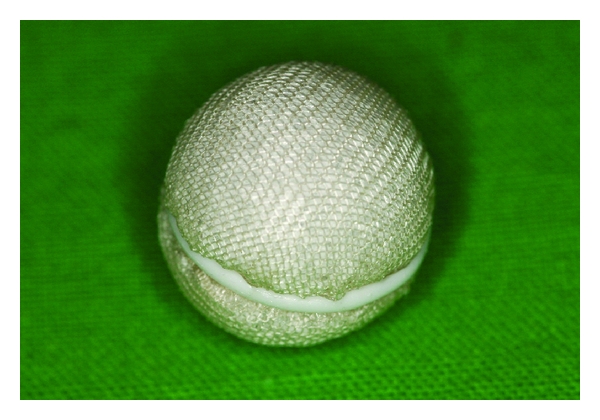
HA orbital implant with the polyglactin 910 mesh covering at the time of implantation.

The authors received the ethical approval of the Semmelweis University to conduct this study and complied with institutional ethical use protocols and the guidelines of the Helsinki Declaration. A total of 30 patients requiring enucleation of the globe were treated with spherical HA and AO orbital implants (France Chirurgie Instrumentation FCI, Issy-Les-Moulineaux, France). The reason for enucleation was intraocular tumor in 10 cases, severe trauma in 10 cases, blind painful eye in 8 cases, microphthalmos in 1 case, and microphthalmos with cyst in 1 case. The diameters of the spherical implants were: 16 mm in one case, 18 mm in 13 cases, and 20 mm in 16 cases (Figures 1 and 2).

Implantation of spherical HA and AO included the following steps. A conjunctival peritomy was performed securing the muscle belly of each of the four rectus muscles with a double-armed suture just behind its insertion on the globe. Then disinsertion of the four rectus muscles was performed sequentially. Then globe was then removed. A set of sizing spheres allowed determination of the size of the orbital implant to be used. Then a spherical HA orbital spherical implant wrapped in polyglactin 910 mesh (Vicryl Mesh Ethicon Inc., Sommerville NJ, USA) or AO was inserted into the muscle cone. The orbital implant was the proper size when it was the largest implant that could be placed into the depth of the orbit. The horizontal and vertical rectus muscles were brought together over the implanted sphere with attachment of the muscles to the Vicryl mesh. The tenon fascia was closed over the muscles and sphere horizontally. Finally the conjunctiva was closed.

In the postoperative phase a firm pressure dressing was used for 4–6 days. Topical antibiotics are applied 4-5 times daily for 4 weeks. The socket was fit with an artificial eye 6–8 weeks postoperatively provided that all oedema had subsided.

### 2.2. Radiographic Methods

Since the numerous methods for porous structure estimations are unsuitable for *in vivo* utilization, the least invasive method using CBCT was used in this study. A large volume CBCT scanner, an iCat Classic (Xoran Technologies, Ann Arbour Michigan, USA) with the following characteristics 120 KV, pixel size: 0.25 mm, slice increment 0.25 mm, FOV 16 cm was used for porous structure evaluation of the 30 patients.

Hounsfield units consist of a linear transformation of the original linear attenuation coefficient measurement into one in which the radiodensity of distilled water at standard pressure and temperature (STP) is defined as zero Hounsfield units (HU), while the radiodensity of air at STP is defined as −1000 HU. 

Definition of Hounsfield units is

(1) $$\text{HU}=\frac{\mu_{X}-\mu_{\text{water}}}{\mu_{\text{water}}-\mu_{\text{air}}}\times 1000.$$

This definition is essential for MSCT scanners which are calibrated with reference to water. 

**Figure 3:**
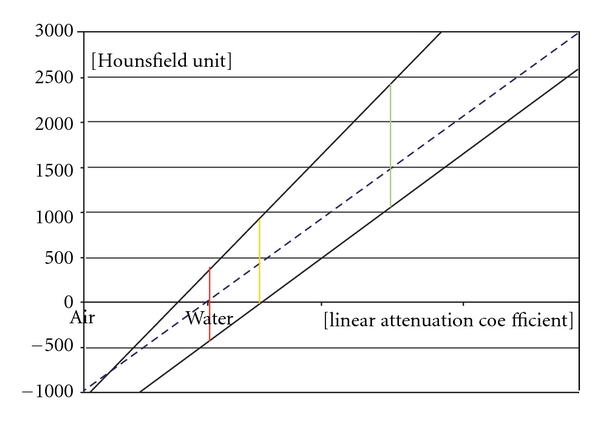
The conventional calibrated (MSCT) and noncalibrated (CBCT) function of the attenuation coefficient and HU. The conventional calibration is represented by an interrupted blue line. The MSCT is calibrated with this method. These functions are different with CBCT scanners as this calibration is lacking. Two examples are shown as noninterrupted black lines.

The CBCT also uses a linear Hounsfield unit scale which is not calibrated with water on a regular basis. Calibrations can be done with two different linear attenuation materials by measuring the HU in the CBCT and MSCT systems (Figure 3).

The greater the difference in the attenuation of the two materials, within the linear function range, the more accurate the calibration is. The HU acquired by the CBCT can be transformed to the conventional MSCT-like HU scale. 

There can be a difference between the linear attenuation coefficient and HU with each CBCT scanning procedure. Therefore each CBCT acquisition required a separate calibration in this study. 

**Figure 4:**
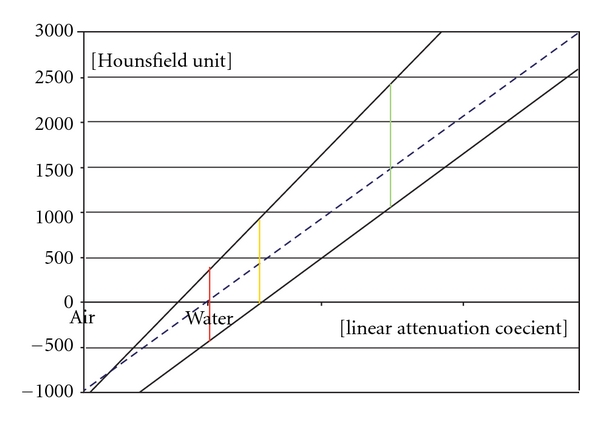
Multiplanar reconstruction (MPR) of the dedicated orbital volume (DV). Axial view.

**Figure 5:**
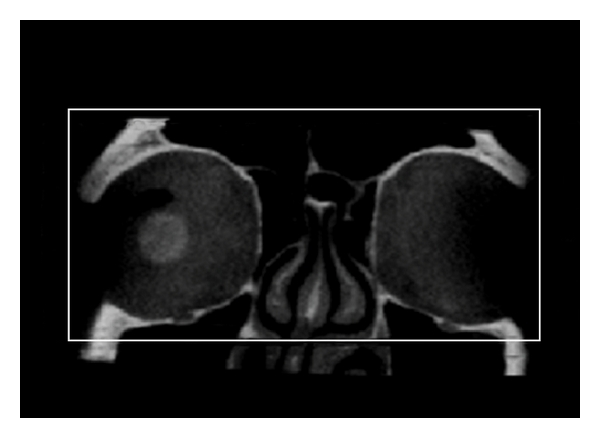
Multiplanar reconstruction (MPR) of the dedicated orbital volume (DV). Coronal view.

**Figure 6:**
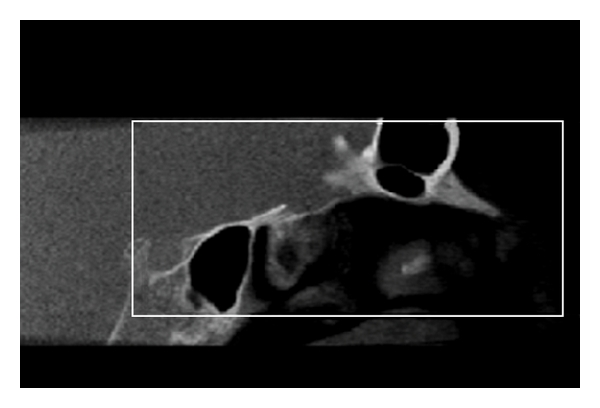
Multi-planar reconstruction (MPR) of the dedicated orbital volume (DV). Sagittal view.

**Figure 7:**
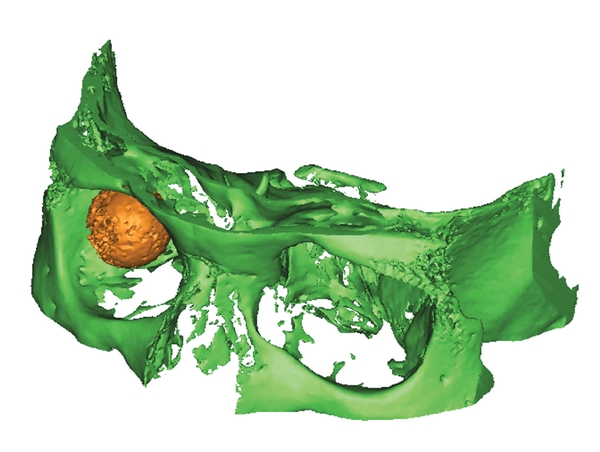
Volume rendering of the dedicated orbital volume (DV). The DV is illustrated (green) and the orbital implant (yellow).

A dedicated volume bordered by the inferior, superior, lateral orbital margins and the posterior part of the sella turcica were separated from the rest of the captured images and termed the dedicated volume (DV) (Figures 4, 5, 6, and 7).

This volume contained decreased CBCT-related artefacts and was the focus of this experiment. The gross volume of the orbital implants was known to be 16, 18, or 20 mm-diameter spheres and the corresponding diameter spherical object used to determine the orbital implant volume (OI).

**Figure 8:**
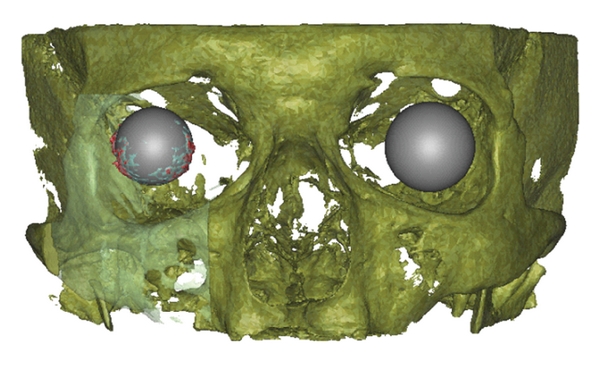
Volume rendering of the scanned volume with the orbital implant and the contralateral corpus vitreum (patient ID 1.27).

For calibration purposes and to correct for orbital implant HU values two objects with different linear attenuation coefficient were chosen from the FOV. The first object was the corpus vitreum of the contralateral orbit, and its isolated volume was referred as the contralateral corpus vitreum volume (CV) (Figure 8).

This was assumed to be a constant density region, close to the density of the water for all patients. The second object for calibration was the maximum HU and maximum linear attenuation coefficient of the dedicated volume of the orbital region.

The mean HU value, apparent density, the OI, and CV, as well as the maximal HU value in the DV were recorded. An independent investigator estimated orbital implant radius diameter with the use of a radius based-3-point fitting sphere drawing method available with Mimics 12 software (Materialise, Leuven, Belgium). The time between the implantation and the date of scanning was also noted.

 A control group was established using ten randomly selected head and neck scans from an archived past data pool of MSCT scans from a General Electric, LightSpeed Ultra MSCT scanner (Baltimore MD, USA) with the following characteristics: 120 KV, 246.35mAs, pixel size 0.326 mm, slice increment 0.625 mm, FOV 16.7 cm. The same standard and bone plus acquisition algorithm were used in all 10 cases. The mean HU value of the CV and the maximal HU value of the DV were checked in the control group within the dedicated orbital region. The radiologic evaluation was performed with Mimics 12, while Excel (Microsoft, Redmond, WA, USA) and SPSS (IBM, Somers NY, USA) programs were used for statistical analysis.

## 3. Results

The results of the 10 head and neck control MSCT scans showed that the mean CV density was 5.93 HU (±3.97 HU) while the DV maximal HU value was 2024.67 HU (±103.49 HU).

The results of the 30 patients' iCAT CBCT scans are shown in Table 1 in which the orange colour represents AlO implants and light blue the HA implants. The ID numbers with light colour indicate the excluded cases. The cases appear in the consecutive order in which they were scanned.

After review of the quality of the CBCT scans by an independent observer, two patients were excluded from the study. One was excluded because of noncoherent scanning FOV due to inadequate head positioning which resulted in poor image quality. The second patient was a trauma patient who was excluded because there was high-density metal nearby causing artefacts beyond the average noise levels. 

## 4. Discussion 

Orbital implants can be classified as porous and nonporous. The porous orbital implants are composed of either HA, porous polyethylene (Medpor), or aluminium [1]. HA orbital implants were introduced by Perry in 1985 [31], and porous polyethylene orbital implants were introduced in 1989. Porous structures allow fibrovascular ingrowth into the implant and integration with orbital tissues [1]. Most porous implants used today are spherical. 

Porous HA can be made by using the xenogenic matrix from a specific genus of reef-building coral [1] and changed from calcium carbonate to calcium phosphate by a hydrothermal exchange reaction. HA can also be completely synthetic. HA constitutes the primary inorganic portion of human bone. The porous form has a microarchitecture similar to human cancellous bone with numerous interconnecting channels with an average diameter of 500 *μ*m [1]. When HA is implanted next to bone, new bone growth occurs within its pores [1]. When HA is implanted within soft tissues, fibrovascular tissue grows into the pores [11, 12, 32]. 

Polyglactin 910 (Vicryl) wrapped HA implants were used in 18 and AlO in 12 patients in this double-blinded study. While the surface of HA implant is very rough, compared to the lightweight more uniform microcrystalline structure of AO [33], wrapping the orbital implant allows attachment of the extraocular muscles. A fibrovascular capsule of variable thickness forms external to the polyglactin mesh and replaces it by 12 weeks. Polyglactin 910 mesh-wrapped HA implants must be placed deeply into the orbit with adequate soft tissue coverage to offset a higher exposure risk [17]. 

Vascularisation of an unwrapped HA implant takes approximately 6 months; however when the HA implant is wrapped with polyglactin mesh the speed of vascularisation is more variable [18]. *In vitro* fibroblasts and osteoblasts proliferate more rapidly on the aluminium oxide compared to HA suggesting the more biocompatible feature [34, 35]. Animal studies of HA implants showed evidence of soft-tissue ingrowth into the material. Shields et al. reported the first histopathologic evidence of fibrovascularisation of this implant in the human orbit and showed that the implant was partially vascularised as early as 4 weeks after placement [32]. 

Gadolinium-enhanced MRI is a well-known method to follow fibrovascular ingrowth into polyethylene orbital implants. MRI showed no influence on the fibrovascular ingrowth into orbital implants whether enucleation or evisceration was performed using an animal study [36]. MRI showed that HA implants took longer to allow fibrovascular ingrowth [15, 16]. A retrospective study of 45 patients with orbital implants who underwent gadolinium-DTPA T1-weighted MR imaging showed homogenous vascularization with an intense enhancement pattern suggesting adequate vascular ingrowth after 5 to 6 months following implantation [14]. Potential implant failure may also be predictable with MRI scanning [37]. 

Bone scanning using 99mTc (technetium) methylene diphosphonate (99mTc-MDP) bone scan is another method to follow fibrovascular ingrowth into orbital implants. Bone scans however show that the orbital implants may be fully penetrated by the 5th to 8th week following implantation [38, 39]. Bone scans may be useful when the effect of growth factors is examined on HA orbital implants or with other scaffolds [40]. Based on bone scan imaging the HA fibrovascularization is more rapid than porous polyethylene implants [41]. Nuclear medicine studies have great potential in future implant and biomaterial evaluation [42]. A single-photon emission computed tomography (SPECT) study showed that increasing pore size of the implant was associated with a higher rate vascular penetration while the amount of fibrotic tissue was decreased. The authors suggested the pore size to be larger than 400 micrometers [43]. Future fusion of these and other imaging modalities may become a reality. In fact MRI has been used in the tissue engineering paradigm to evaluate angiogenesis in certain scaffolds [44]. At the present time MRI may be the best modality to assess angiogenesis and fibrovascular ingrowth but CBCT is simpler, without the need for intravenous dye injection and with easier to install equipment. 

There are two major advantages in using fibrovascularised implants in the orbit. The implant is less likely to extrude because it becomes biologically fixed in the soft tissues of the orbit. The implant is theoretically less likely to become infected since the implant is incorporated with host blood vessels within the recipient site with improved access to the host immune defences [17]. 

In this study the MSCT control group was used to determine the maximal HU of the DV (2024.7 HU ± 103.49 HU) and the mean density of CV (5.93 HU ± 3.97 HU) which correspond to the CBCT. The CBCT calibrations were performed within each scan with the CV mean and for the DV maximum HU values as two well-defined density values corresponding to the control group. 

**Figure 9:**
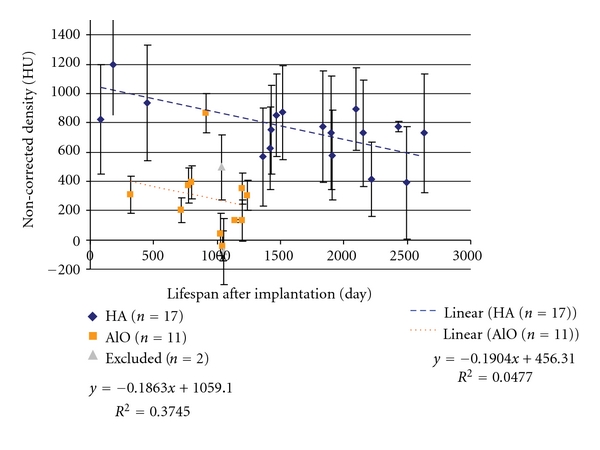
Scatter plot of the time between implantation and the HU (density). Conventionally noncalibrated and noncorrected plots. Linear regression was applied to both implants separately. The excluded (*n* = 2) cases are also indicated.

This unique set of patients with implantation times ranging from 79 to 2636 days provides an opportunity to study the evolution of HUs of the implants over time (Figure 9). 

Correction equation for the noncalibrated values is

(2) $$\begin{array}{cc} {\text{HU}}_{\text{corrected}} & =\frac{{\text{HU}}_{\text{oi}\;\text{DVT}}-{\text{HU}}_{\text{cv}\;\text{DVT}}}{{\text{HU}}_{\text{dv}\;\text{DVT}}-{\text{HU}}_{\text{cv}\;\text{DVT}}}\\ & \;\times \left({\text{HU}}_{\text{dv}\;\text{MSCT}}-{\text{HU}}_{\text{cv}\;\text{MSCT}}\right)+{\text{HU}}_{\text{cv}\;\text{MSCT}}. \end{array}$$

**Figure 10:**
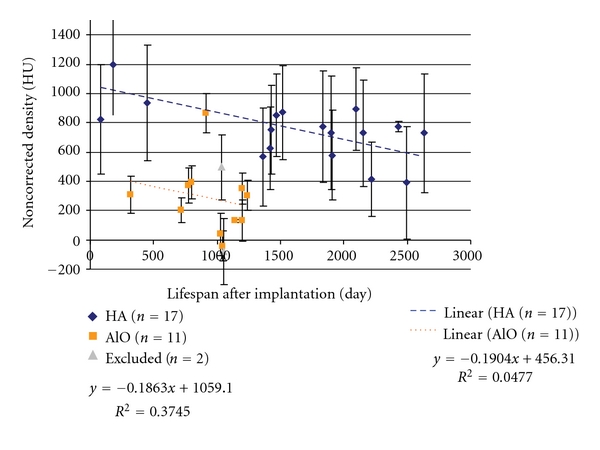
Scatter plot of the time between implantation and the HU (density) with conventionally noncalibrated and corrected plots.

The apparent density (HU) data from the remaining 28 implants (HA *n* = 17, AO *n* = 11) was plotted versus the life span of the implant (days following implantation). Linear regression was used to estimate the changes during this period. The regression equations for both are indicated on the plotted Figure 10. 

The following trend was observed based on the plots. The HA implants show a corrected 10 HU decrease while AlO decreases 14 HU on average on a yearly basis. Interpreting these results requires discrimination between the high apparent density (HU) of the implant materials and the low apparent density of the surrounding and infiltrating fibrovascular structures. Taking this into account the decrease in HU levels over the gross volume may represent the maturation, transformation of the fibrovascular ingrowth into the remaining porous orbital implant or migration and possibly resorption of the implant material in small portions. 

**Figure 11:**
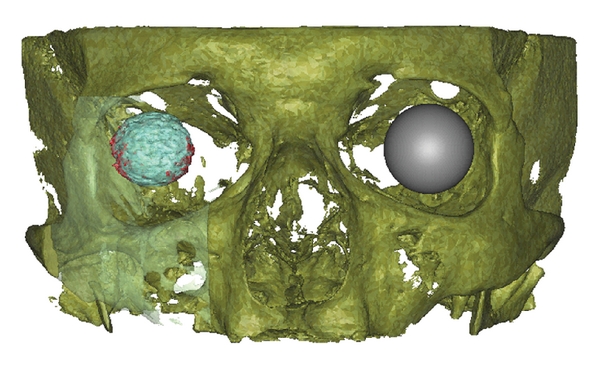
Frontal view of volume rendering of the scanned volume with the orbital implant and the contralateral corpus vitreum. The orbital implant volume within the factory implant diameter (light blue) and beyond the factory implant diameter (red). The contralateral orbit is represented as a gray sphere (patient ID 1.27).

**Figure 12:**
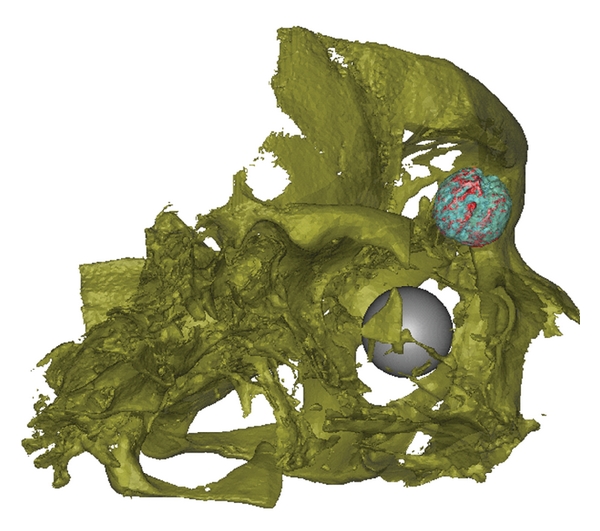
Oblique lateral view of volume rendering of the scanned volume with the orbital implant and the contralateral corpus vitreum. The orbital implant volume within (light blue) and beyond (red) the factory implant diameter. The contralateral orbit is represented as a gray sphere (patient ID 1.27).

 Parts of the orbital implant which exceed the original diameter of the implant were observed. There are spikes around the marginal region of the orbital implant with different amounts in the lower nasal and temporal direction (Figures 11 and 12). It is not clear if this is the part of the orbital implant or a CBCT-related artefact. 

Orientation, position, and bulk volume estimation are possible with CBCT; however net volume measurements were not made with settings used by the authors, because the resolution of the acquisition is unable to detect the smaller details of this structure. 

 The estimated diameter based on the 3-point fitting sphere drawing method showed a 0.2839 mm standard deviation compared to the manufacturer's original value. This is within the range of the actual acquisition technique resolution. 

Since this is an *in vivo* study and none of the samples required removal, there were no samples for decalcification or histological evaluation. 

## 5. Conclusions 

The major goals of enucleation are to remove a diseased globe which is intact and to provide the patient with cosmetically acceptable appearance. HA orbital implants can be used for long-term volume replacement after orbital enucleation. 

The general trend with decreasing implant density with time is noted with both HA and AO implants populations with and without HU correction, although the correction makes the HU values more consistent. This tendency was not confirmed histologically because there were no failures requiring removal of any of the orbital implants, leaving no ethical way to biopsy or sample the surround orbital soft tissues or the implants. Further studies in animals are required to confirm these finding with histological samples and a highly standardized CBCT protocol. 

The CBCT acquisition technique is attractive due to its lower radiation exposure when compared to MSCT for long-term orbital prosthesis monitoring. Such long-term monitoring may require serial scanning of the same patient. CBCT is a noninvasive imaging modality suitable for implant evaluation in terms of orientation, position, bulk volume, and gross density without the possibility of net volume estimation. This CBCT study has provided a minimally invasive platform for the future long-term evaluation of porous calcified implants and has provided some insights into the long-term behaviour of hybrid polyglactin HA orbital prosthesis. 

## Figures and Tables

**Table 1:** 

	Orbital implant						Corpus vitreum	Isolated volume
ID number	Diameter [mm]		Lifespan (days)	Volume mean (HU)	Volume mean SD (HU)	Corrected mean (HU)	Volume mean (HU)	Volume maximum (HU)
	Measured	Estimated						
1.1	18	17,96	1051	**−79**	226	**345**	−425	1606
1.2	20	20,5	1420	**623**	281	**754**	−209	2006
1.3	20	19,96	1037	**496**	222	**496**	−344	3071
1.4	18	17,66	1361	**566**	337	**713**	−179	1920
1.5	20	20,5	1040	**−42**	101	**434**	−458	1476
1.6	18	16,26	2493	**391**	383	**598**	−147	1664
1.7	20	19,68	1911	**579**	304	**825**	−331	1884
1.8	20	19,58	1031	**38**	143	**480**	−446	1588
1.9	20	20,14	776	**371**	121	**411**	−87	2162
1.10	20	20,1	718	**200**	85	**397**	−198	1828
1.11	18	17,92	1196	**350**	105	**360**	−39	2146
1.12	20	20,1	1466	**852**	281	**939**	−104	1939
1.13	18	17,86	1831	**773**	383	**784**	−187	2273
1.14	18	17,4	317	**308**	128	**417**	−131	1995
1.15	18	17,88	1425	**750**	303	**891**	−127	1848
1.16	20	20,08	79	**823**	376	**779**	−131	2329
1.17	18	18,56	2151	**728**	361	**790**	−166	2106
1.18	18	17,92	2427	**775**	37	**696**	−78	2384
1.19	20	19,78	1237	**302**	102	**424**	−148	1995
1.20	20	20,26	1202	**132**	143	**321**	−212	1958
1.21	20	19,62	213	**1432**	521	**786**	326	3150
1.22	18	17,62	2219	**414**	253	**695**	−285	1736
1.23	20	19,72	1140	**131**	11	**363**	−228	1773
1.24	18	17,96	1903	**729**	388	**810**	−166	2051
1.25	16	17,78	2636	**729**	407	**711**	−77	2200
1.26	10	19,66	801	**390**	112	**387**	−29	2162
1.27	20	20,4	452	**935**	397	**702**	78	2532
1.28	20	20,7	1515	**871**	322	**825**	−57	2200
1.29	20	19,78	910	**865**	131	**378**	340	3150
1.30	20	20,4	2093	**893**	279	**807**	40	2161
